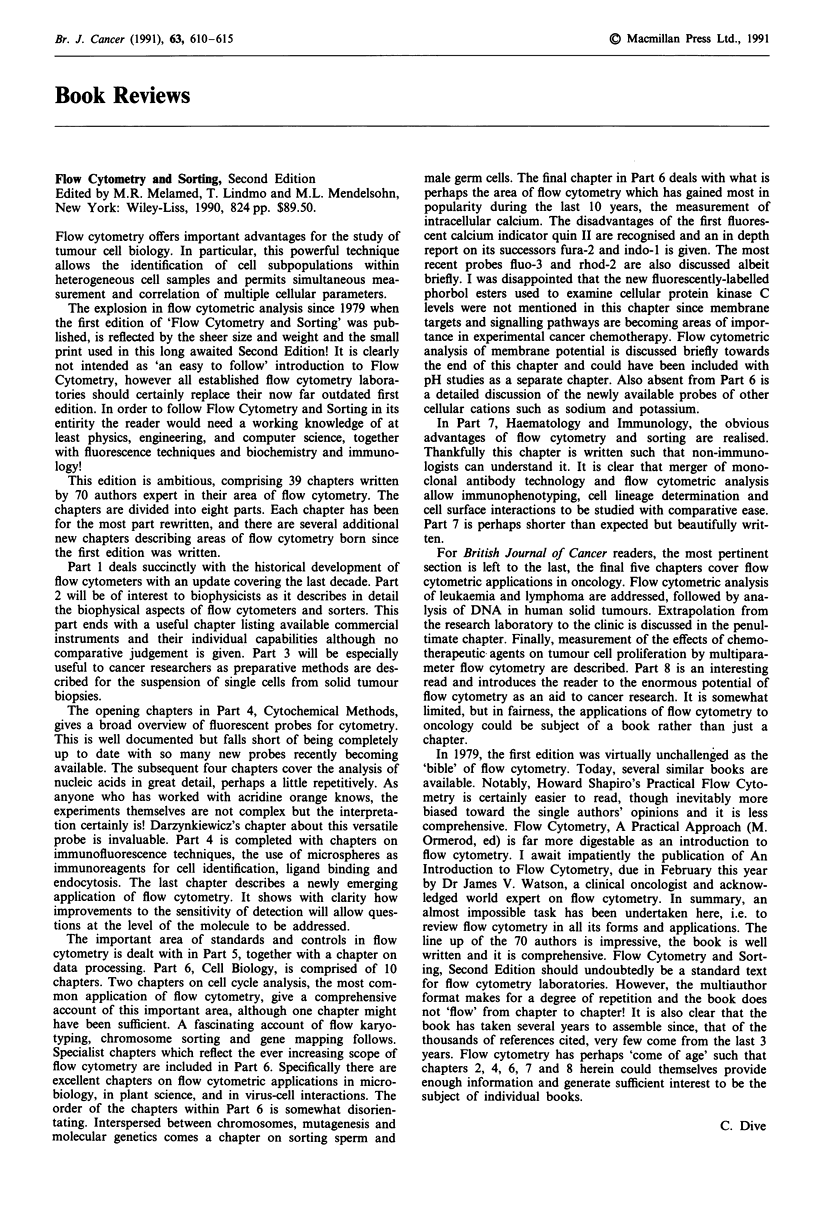# Flow Cytometry and Sorting

**Published:** 1991-09

**Authors:** C. Dive


Br. J. Cancer (1991), 63, 610-615 © Macmillan Press Ltd., 1991

### Book Reviews

**Flow Cytometry and Sorting**, Second Edition
Edited by M.R. Melamed, T. Lindmo and M.L. Mendelsohn, New York: Wiley-Liss, 1990, 824 pp. $89.50.

Flow cytometry offers important advantages for the study of tumour cell biology. In particular, this powerful technique allows the identification of cell subpopulations within heterogeneous cell samples and permits simultaneous measurement and correlation of multiple cellular parameters.

The explosion in flow cytometric analysis since 1979 when the first edition of 'Flow Cytometry and Sorting' was published, is reflected by the sheer size and weight and the small print used in this long awaited Second Edition! It is clearly not intended as 'an easy to follow' introduction to Flow Cytometry, however all established flow cytometry laboratories should certainly replace their now far outdated first edition. In order to follow Flow Cytometry and Sorting in its entirity the reader would need a working knowledge of at least physics, engineering, and computer science, together with fluorescence techniques and biochemistry and immunology!

This edition is ambitious, comprising 39 chapters written by 70 authors expert in their area of flow cytometry. The chapters are divided into eight parts. Each chapter has been for the most part rewritten, and there are several additional new chapters describing areas of flow cytometry born since the first edition was written.

Part 1 deals succinctly with the historical development of flow cytometers with an update covering the last decade. Part 2 will be of interest to biophysicists as it describes in detail the biophysical aspects of flow cytometers and sorters. This part ends with a useful chapter listing available commercial instruments and their individual capabilities although no comparative judgement is given. Part 3 will be especially useful to cancer researchers as preparative methods are described for the suspension of single cells from solid tumour biopsies.

The opening chapters in Part 4, Cytochemical Methods, gives a broad overview of fluorescent probes for cytometry. This is well documented but falls short of being completely up to date with so many new probes recently becoming available. The subsequent four chapters cover the analysis of nucleic acids in great detail, perhaps a little repetitively. As anyone who has worked with acridine orange knows, the experiments themselves are not complex but the interpretation certainly is! Darzynkiewicz's chapter about this versatile probe is invaluable. Part 4 is completed with chapters on immunofluorescence techniques, the use of microspheres as immunoreagents for cell identification, ligand binding and endocytosis. The last chapter describes a newly emerging application of flow cytometry. It shows with clarity how improvements to the sensitivity of detection will allow questions at the level of the molecule to be addressed.

The important area of standards and controls in flow cytometry is dealt with in Part 5, together with a chapter on data processing. Part 6, Cell Biology, is comprised of 10 chapters. Two chapters on cell cycle analysis, the most common application of flow cytometry, give a comprehensive account of this important area, although one chapter might have been sufficient. A fascinating account of flow karyotyping, chromosome sorting and gene mapping follows. Specialist chapters which reflect the ever increasing scope of flow cytometry are included in Part 6. Specifically there are excellent chapters on flow cytometric applications in microbiology, in plant science, and in virus-cell interactions. The order of the chapters within Part 6 is somewhat disorientating. Interspersed between chromosomes, mutagenesis and molecular genetics comes a chapter on sorting sperm and male germ cells. The final chapter in Part 6 deals with what is perhaps the area of flow cytometry which has gained most in popularity during the last 10 years, the measurement of intracellular calcium. The disadvantages of the first fluorescent calcium indicator quin II are recognised and an in depth report on its successors fura-2 and indo-1 is given. The most recent probes fluo-3 and rhod-2 are also discussed albeit briefly. I was disappointed that the new fluorescently-labelled phorbol esters used to examine cellular protein kinase C levels were not mentioned in this chapter since membrane targets and signalling pathways are becoming areas of importance in experimental cancer chemotherapy. Flow cytometric analysis of membrane potential is discussed briefly towards the end of this chapter and could have been included with pH studies as a separate chapter. Also absent from Part 6 is a detailed discussion of the newly available probes of other cellular cations such as sodium and potassium.

In Part 7, Haematology and Immunology, the obvious advantages of flow cytometry and sorting are realised. Thankfully this chapter is written such that non-immunologists can understand it. It is clear that merger of monoclonal antibody technology and flow cytometric analysis allow immunophenotyping, cell lineage determination and cell surface interactions to be studied with comparative ease. Part 7 is perhaps shorter than expected but beautifully written.

For *British Journal of Cancer* readers, the most pertinent section is left to the last, the final five chapters cover flow cytometric applications in oncology. Flow cytometric analysis of leukaemia and lymphoma are addressed, followed by analysis of DNA in human solid tumours. Extrapolation from the research laboratory to the clinic is discussed in the penultimate chapter. Finally, measurement of the effects of chemotherapeutic agents on tumour cell proliferation by multiparameter flow cytometry are described. Part 8 is an interesting read and introduces the reader to the enormous potential of flow cytometry as an aid to cancer research. It is somewhat limited, but in fairness, the applications of flow cytometry to oncology could be subject of a book rather than just a chapter.

In 1979, the first edition was virtually unchallenged as the 'bible' of flow cytometry. Today, several similar books are available. Notably, Howard Shapiro's Practical Flow Cytometry is certainly easier to read, though inevitably more biased toward the single authors' opinions and it is less comprehensive. Flow Cytometry, A Practical Approach (M. Ormerod, ed) is far more digestable as an introduction to flow cytometry. I await impatiently the publication of An Introduction to Flow Cytometry, due in February this year by Dr James V. Watson, a clinical oncologist and acknowledged world expert on flow cytometry. In summary, an almost impossible task has been undertaken here, i.e. to review flow cytometry in all its forms and applications. The line up of the 70 authors is impressive, the book is well written and it is comprehensive. Flow Cytometry and Sorting, Second Edition should undoubtedly be a standard text for flow cytometry laboratories. However, the multiauthor format makes for a degree of repetition and the book does not 'flow' from chapter to chapter! It is also clear that the book has taken several years to assemble since, that of the thousands of references cited, very few come from the last 3 years. Flow cytometry has perhaps 'come of age' such that chapters 2, 4, 6, 7 and 8 herein could themselves provide enough information and generate sufficient interest to be the subject of individual books.

C. Dive